# “Now is the time for institutions to be investing in growing exercise programs as part of standard of care”: a multiple case study examining the implementation of exercise oncology interventions

**DOI:** 10.1007/s00520-023-07844-x

**Published:** 2023-06-26

**Authors:** Louise Czosnek, Nicole M. Rankin, Prue Cormie, Andrew Murnane, Jane Turner, Justin Richards, Simon Rosenbaum, Eva M. Zopf

**Affiliations:** 1 Mary MacKillop Institute for Health Research, Australian Catholic University, Melbourne, Victoria Australia; 2 Faculty of Medicine and Health, University of Sydney, Sydney, New South Wales Australia; 3 Faculty of Medicine, Dentistry and Health Sciences, University of Melbourne, Melbourne, Victoria Australia; 4 Peter MacCallum Cancer Centre, Melbourne, Victoria Australia; 5 Sir Peter MacCallum Department of Oncology, The University of Melbourne, Melbourne, Victoria Australia; 6 Concord Repatriation General Hospital, Concord, New South Wales Australia; 7 Faculty of Health, Victoria University of Wellington, Wellington, New Zealand; 8 Discipline of Psychiatry and Mental Health, University of New South Wales, Sydney, Australia; 9 School of Health Sciences, University of New South Wales, Sydney, New South Wales Australia; 10 Cabrini Cancer Institute, Department of Medical Oncology, Cabrini Health, Melbourne, Victoria Australia

**Keywords:** Exercise, Implementation, Cancer, Physical activity, Evaluation

## Abstract

**Background:**

Implementation science seeks to systematically identify determinants, strategies, and outcomes within a causal pathway to help explain successful implementation. This process is applied to evidence-based interventions (EBIs) to improve their adoption, implementation, and sustainment. However, this method has not been applied to exercise oncology services, meaning we lack knowledge about implementing exercise EBIs in routine practice. This study aimed to develop causal pathways from the determinants, strategies (including mechanism of change), and implementation outcomes to explain exercise EBIs implementation in routine cancer care.

**Methods:**

A multiple-case study was conducted across three healthcare sites in Australia. Sites selected had implemented exercise within routine care for people diagnosed with cancer and sustained the delivery of services for at least 12 months. Four data sources informed the study: semi-structured interviews with staff, document reviews, observations, and the Program Sustainability Assessment Tool (survey). Framework analysis was applied to understand the findings. The Implementation Research Logic Model was used to identify commonalities in implementation across sites and develop causal pathways.

**Results:**

Two hundred and eighteen data points informed our findings. Across sites, 18 determinants and 22 implementation strategies were consistent. Sixteen determinants and 24 implementation strategies differed across sites and results of implementation outcomes varied. We identified 11 common pathways that when combined, help explain implementation processes. The mechanisms of implementation strategies operating within the pathways included (1) knowledge, (2) skills, (3) secure resources, (4) optimism, and (5) simplified decision-making processes associated with exercise; (6) relationships (social and professional) and support for the workforce; (7) reinforcing positive outcomes; (8) capability to action plan through evaluations and (9) interactive learning; (10) aligned goals between the organisation and the EBI; and (11) consumer-responsiveness.

**Conclusion:**

This study developed causal pathways that explain the how and why of successful implementation of exercise EBIs in cancer care. These findings can support future planning and optimisation activities by creating more opportunities for people with cancer to access evidence-based exercise oncology services.

**Implications for cancer survivors:**

Understanding how to implement exercise within routine cancer care successfully is important so cancer survivors can experience the benefits of exercise.

**Supplementary Information:**

The online version contains supplementary material available at 10.1007/s00520-023-07844-x.

## Introduction

Medical advances in cancer screening, diagnosis and treatment mean people are living longer after a cancer diagnosis [1, 2]. As life expectancy increases, efforts to optimise the quality of a longer life are critical. Exercise is an evidence-based intervention (EBI) increasingly employed across the cancer care continuum [3]. Exercise is applied to prevent cancer, better prepare people for cancer treatments, ameliorate the disease sequela associated with its treatment and improve life after a cancer diagnosis [4, 5].

Despite a substantial evidence base and recommendations within clinical practice guidelines [4, 6, 7], exercise is not routinely integrated into cancer care during and after treatment completion [8]. This is not entirely unexpected, as successful implementation of EBIs in healthcare is notoriously difficult, with many complex factors at the patient, provider, organisational and health system levels influencing uptake [9–11]. The methods employed in the discipline of implementation science are used to improve understanding and help explain the outcomes and success of the implementation process [12].

Implementation science applies a sequential and structured approach to produce generalisable knowledge [13]. Developing generalisable knowledge encourages the replication of critical findings across sites/context to help spread effective EBIs. It can also be applied prospectively to understand the extent to which results are transferable to other settings (i.e., transferability of findings) [14]. Several implementation science constructs are recognised and enable transferability, including (1) determinants (i.e., barriers and facilitators) that shape the contextual environment and influence the implementation process [15, 16]; (2) implementation strategies, which are the actions applied to augment the contextual environment and create favourable conditions for implementation [17–19]; (3) implementation outcomes of these efforts to define and measure whether successful implementation was achieved [20, 21]; and (4) mechanisms of change, which are the “processes or events through which an implementation strategy operates to affect desired implementation outcomes” [22]. Implementation research has tended to study these constructs in isolation; however, greater emphasis is now focused on the combined analyses to explain how these constructs operate together [23]. Combined analysis can include sequentially linking these constructs to elucidate the explanatory causal pathway and hypothesised mechanisms contributing to successful implementation [24–27]. Without this combined analysis, the ability to understand or explain the implementation process and how impact can be achieved on a larger scale is compromised [23].

In cancer care settings, the determinants of exercise EBIs are well studied [28–31]. A recent scoping review systematically identified 243 barriers to implementing exercise oncology EBIs, including limited time during consultations, dedicated exercise resources, and funding [32]. Studies of implementation strategies in exercise oncology are less common [33–36], despite a recent review identifying that using implementation strategies resulted in greater uptake of exercise than if these strategies were absent [37]. Evaluations of implementation outcomes have shown mixed results in understanding what constitutes success [38–41]. To our knowledge, only one study in exercise and cancer has sought to explain the mechanisms for how an implementation strategy is proposed to enact the change function [42]. Research on exercise EBIs in cancer care would benefit from integrating these constructs within a research study to improve targeted implementation efforts and support transferability to increase impact at scale. This study aimed to address this gap by developing explanatory causal pathways for implementing exercise in routine cancer care. We aimed to systematically identify the determinants, implementation strategies (including mechanisms of change), and implementation outcomes for exercise EBIs in cancer care based on established implementation science frameworks [15, 17, 20]. We used a multiple case study design to elucidate commonalities in pathways across cancer healthcare sites. The constructs were linked using the Implementation Research Logic Model (IRLM) [26] to produce the causal pathways.

By testing this approach, we sought to identify the transferable elements that could be relevant in future implementation efforts. The specific objectives of the study were to:

- Identify the commonalities and differences in determinants, implementation strategies and implementation outcomes (acceptability, fidelity, penetration, and sustainability) across exercise oncology services
- Develop an explanatory causal pathway for the implementation processes from the common elements that exist across case sites

## Method

### Study design and participating sites

The methods and theoretical application of this study have been previously described [43]. Briefly, a multiple case study [44] of implementation was conducted at three healthcare sites across New South Wales and Victoria, Australia. Sites had implemented exercise within routine care for people diagnosed with cancer and sustained service delivery for at least 12 months. We examined the exercise EBIs that were operating at each site.

### Case descriptions

#### Case site A

Case site A is a publicly funded healthcare facility in New South Wales (NSW), Australia. It delivers specialist clinical services across various disciplines including cardiology, mental health, orthopaedics, and oncology. The organisation is also an established learning and teaching institute with research affiliations. The exercise EBI is delivered through the cancer survivorship service. The survivorship service was established in 2013 and is accessible to anyone who undergoes active cancer treatment at the site. Upon entry to the service, patients undergo an initial review with members of a multi-disciplinary team that includes an oncologist, nurse, psychologist, dietitian, and accredited exercise physiologist (AEP). The multi-disciplinary team then develops a holistic treatment plan. Multiple services are accessible through the survivorship program including individual consultations with allied health professionals, community-based group exercise programs, or participation in classes and workshops. Classes include yoga, art therapy, meditation, QiGong, and scrapbooking. Patients who undergo an initial assessment with the survivorship team can continue their medical treatment with the survivorship service or return to their original medical team for ongoing care. Approximately one-third of patients continue their care with the survivorship team for up to 5 years.

#### Case site B

Case site B is a community-based not-for-profit organisation in Victoria, Australia. The service commenced as a research project funded by the Victorian State Government. Through the initial funding, a company that continues to deliver exercise EBIs for people with and living after a cancer diagnosis was established. The service operates as a user-pay model that subsidises 50% of the EBI costs through donations/fund-raising and, where possible, accessing the universal healthcare system in Australia (Medicare). Case site B delivers the exercise EBI at five locations across metropolitan Melbourne (Victoria, Australia). Several factors were considered in selecting sites, including accessibility, ambience, and amenities. That is close to public transport, car parking and a coffee shop and facilities to conduct private clinical assessments and opportunities to participate in different exercise modalities. The delivery sites are owned and operated by third parties. People access the service through self-referral or referral from sources such as their workplace, health insurer, or medical team. On referral, an initial assessment is completed by an AEP and an individualised exercise plan is developed.

#### Case site C

Case site C is a publicly funded healthcare facility established with the sole purpose of treating cancer. The main campus is in metropolitan Melbourne, with four satellite sites located in local neighbourhoods and regional areas across Victoria, Australia. Case site C delivers adult cancer services through 13 cancer streams and operates a dedicated youth service. Exercise EBIs are provided for both adults and youth via different service models. For adults, the exercise EBI is situated within a multi-disciplinary prehabilitation program (i.e., a program that focuses on improving physical, emotional, nutritional, and general health before patients commence cancer treatment) and as a standalone allied health service that inpatients and outpatients can access. The prehabilitation program includes a comprehensive assessment and an established care plan that contains interventions from various disciplines (i.e., psychology, dietetics, AEP). The stand-alone allied health service typically offers limited sessions and supports on-referral to exercise opportunities in the community where possible. In the youth service, the exercise EBI is delivered by an AEP who is part of a multi-disciplinary team providing for the health and well-being of youth during and in the years after a cancer diagnosis.

Supplementary file 1 details the exercise EBI delivered at each site.

#### Data sources

Four data sources informed the explanation of implementation: [1] in-depth semi-structured interviews with purposely selected staff; [2] observational visits to the healthcare sites; [3] review of organisational documents; and [4] a validated survey that assesses the EBIs capacity for sustainability (Program Sustainability Assessment Tool (PSAT)) [45]. An interview guide was developed to help focus inquiry through the semi-structured interviews. Staff selected for the interviews represented a cross-section of roles (i.e., delivery staff, organisational executive) to enable comprehensive formation of the implementation process at each site. Observational visits focused on observing how the exercise EBI was delivered within the broader context of the healthcare sites typical operations. The research team asked questions and sought clarification of what was being observed, however, did not interrupt typical exercise programming. Organisational documents sought for review included items such as program-specific protocols, administrative documents, and consultancy reports. A list of typical documents sought was provided to sites by the research team to help identify relevant documents. Finally, the PSAT measures sustainability across eight domains (i.e., environmental support, funding stability, partnerships, organisational capacity, program evaluation, program adaption, communications, and strategic planning) and provides insights into EBIs’ strengths and weaknesses. A sub-set of staff who participated in interviews also completed the PSAT, aligned with the tools recommended use [46, 47]. A case study database was maintained to house and organise data.

#### Implementation science frameworks and program logic

Three frameworks and a program logic were applied to guide different aspects of the study. The Consolidated Framework for Implementation Research (CFIR) was used to identify and prioritise determinants at each site [15]. The strength and valance coding of the CFIR guided prioritisation [48]. The Expert Recommendations for Implementing Change (ERIC) was the taxonomy applied to provide a consistent description of implementation strategies used at each site [17] and the Implementation Outcomes Framework was used to define the outcomes of interest for this study (acceptability, fidelity, penetration, and sustainability) [20]. As the study was concerned with implementation in routine practice, we selected outcomes recommended for measurement in the later stages of implementation [20]. The program logic used to link these frameworks was the IRLM [26].

Supplementary file 2 summarises the conceptual and measurement framework of the study.

#### Data analysis

Descriptive statistics were calculated for the PSAT using IBM SPSS Statistics for Windows, Version 28 [49] to obtain one measure of sustainability per site. These findings were uploaded into NVivo software Version 12 [50], together with other data sources, and framework analysis was undertaken to make sense of the data [51]. Framework analysis includes 5 stages (i.e., familiarisation, identifying themes, indexing, charting, and mapping and interpretation), with the approach to data analysis shifting between deductive and inductive reasoning [52]. A deductive approach was adopted through earlier stages when data was indexed and charted directly to the elements of the CFIR, ERIC and Implementation Outcomes frameworks. Analysis shifted to an inductive approach when the IRLM was used to map and interpret the findings and produce a simplified logic model for each site that reflected the prioritised determinants. Finally, the IRLM provided the architecture for the cross-case analysis. The mechanisms of the implementation strategies were identified with reference to relevant literature [16, 53, 54]. The final step in the analysis was to combine each simplified IRLMs into one logic model by drawing out the common elements through inductive reasoning that contributed to the successful implementation of the exercise EBIs across case sites.

## Results

### Data sources

We collected 218 data points to inform the findings, which included 18 semi-structured interviews, approximately 95 hours of observations, 13 responses to the PSAT and 92 document reviews. Table 1 provides a breakdown of data points across case sites.

**Table 1:** Data sources accessed at each case site

Data sources	Case site A	Case site B	Case site C
*Interview*			
Accredited exercise physiologists (delivery staff)	1	2	3
Program manager	1	1	2
Referral source	1	1	1
Other allied health staff	1	0	2
Executive	1	1	0
**Total**	**5**	**5**	**8**
*Observational visits*			
Number of hours spent onsite	40	40	15
**Total**	**40**	**40**	**15**
*Survey (Program Sustainability Assessment Tool)*			
Number completed at each case site	3	4	6
**Total**	**3**	**4**	**6**
*Documents*			
Program-specific protocols (i.e., exercise templates)	9	3	19
Consultancy reports (i.e., workforce reports)	2	0	0
Summaries of program achievements (i.e., formal and informal evaluations)	2	5	4
Public-facing documentation (i.e., website, newsletter, strategic plans)	10	4	3
Administrative documents (i.e., staff training, funding, position description, meeting minutes)	10	16	5
**Total**	**33**	**28**	**31**
**Overall total**	**81**	**77**	**60**

The bold is used to indicate the total for each section in the table

### Determinants

The number of determinants ranged from 33 to 44 across sites. We identified 18 determinants that were thematically consistent across sites. Most (88.9%, *n*=16) were categorised as facilitators of implementation, and two (11.1%) determinants were categorised as barriers to implementation efforts. Table 2 provides a summary of the 18 determinants that were similar across sites.

**Table 2:** Descriptive example of consistent determinants identified across sites using the Consolidated Framework for Implementation Research

Domain	Construct	Description of determinant	Example
Intervention	Evidence strength and quality (E)	Research is used to build buy-in and support for exercise EBIs.	*At (case site name) exercise is an adjunct therapy embedded in standard cancer care. We follow the COSA position statement that recommends patients with a history of cancer should avoid inactivity and:* *Engage in at least 150 min of moderate intensity or 75 min of vigorous intensity aerobic exercise per week (jogging cycling swimming)* *engage in 2–3 resistance exercise sessions per week, targeting major muscle groups (i.e. lifting weights) —* (DR-33)
	Relative advantage (E)	Over time the perceived benefits of exercise have grown and it is seen as the *most effective* treatment for many side effects of cancer treatment.	*“It’s a bit of a panacea to everything”* (Int-4)
	Adaptability (E)	The workforce adapts: - The exercise EBI to suit patient preferences; and - The referral process to suit referral sources needs	*“it (exercise) is just an evolving thing. We work with the clinicians and how they like to work and I think that is how we (are) successful….”* (Int-1) *“originally, we didn’t run group programs, we used to run individual sessions…one of the biggest changes was with the duration of the intervention…”* (Int-2)
	Trialability (E)	The exercise EBI commenced through a research trial.	*“It is slightly different now because originally it was a research trial.”* (Int-6)
Outer setting	Patient need and resourcing (E)	The mechanisms exist to embed the patient voice within EBI design and delivery (i.e., through advisory groups and satisfaction surveys). This includes being responsive to patient direction and making adaptions to the EBI (see *adaptability*).	*“we established a youth advisory board…who come together to advise us on all issues to do with… cancer, not just for us as a service, but they also report up through to State government. So they're actually advising and guiding on policy as well.”* (Int-14)
	Cosmopolitan (E)	The organisation develops relationships with other organisations to build capacity of the exercise EBI (i.e., on referral of patients when the service reaches capacity).	*“So, we do have a longstanding relationship with a certain institution…they had a common interest in cancer and then from there, they had the capacity to take more referrals over.”* (Int-2)
	External policy and incentives (E)	The COSA position statement on cancer and exercise helped validate the role of exercise in standard cancer care. State government policies reference exercise, which provides strategic policy alignment.	*“The reason that we could establish it was because of the policy of the (State government name) cancer policy to support survivorship. I would say that certainly helped us (and) the COSA position statement and the advocacy and awareness that have come around that.”* (Int-9)
Inner setting	Networks and communications (E)	Strong connections existed between individual staff and within teams that facilitated efficiencies in working relationships and implementation (i.e., corridor conversations, call/email (in place of following formalised procedures)).	*“The other thing is having a personal relationship is actually good. Yeah, we work as a team. I think we know each other pretty well.”* (Int-5)
	Implementation climate (E)	Organisations expect innovation and to be a leader in cancer, which allowed the exercise EBI to grow and transform, despite difficult conditions.	*“I think it can be challenging implementing innovative programs in a time of austerity. With that said I think there is a lot of passion and commitment.”* (Int-1)
	Compatibility (E)	Systems are implemented (i.e., opt-out referral, IT system coordination and EMR) to ensure exercise EBIs fit and are aligned with existing workflows.	*“We have an opt-out model of care…. so everyone that gets referred into the service will be offered exercise physiology throughout the course of their treatment.”* (Int-11)
	Learning climate (E)	Healthcare staff feels supported to seek out new and better ways to integrate evidence/learning in routine practice.	*“There is no expectation that you know, everything. You know, we want to be learning and growing together as a team to make sure that we can deliver the best quality service.”* (Int-9)
	Available resources (B)	Healthcare providers lack of time, which is a byproduct of lack of funding, is a barrier to growing/optimising the exercise EBI.	“*We need more, we need more space, we need more (AEPs), we need more time, we don’t think enough of that…. we could do with more admin support to help with programs that we are all running.”* (Int-4)
	Access to knowledge and information (E)	Healthcare providers aim to create a one-stop shop for exercise and cancer. This means referral sources have easy access to the information they need.	“*I guess I found it difficult to know who to refer to….and to try and find people to refer to is actually quite hard. And so I'd refer people to (organisation name)…. its got a website... and then it's done, it's very quick. I don't have to send an e-mail or anything like that, it's done then and there.”* (Int-10)
Individual	Self-efficacy (E)	Healthcare providers are confident to raise and discuss exercise with patients, akin to how they talk about other treatments. Albeit there are *laggards* who don’t see it as their role.	*“So, a lot of the patients… they are very surprised to hear me talking about exercise when they have just been diagnosed with say breast cancer…. And I am sort of saying well actually there is all this evidence and I have seen patients (with) … very similar cancer and treatments that you’re having and I am going to start prescribing exercise with chemotherapy.”* (Int-4)
	Individual stage of change (B)	Providers are aware of the value of exercise, but they do not routinely act to discuss/refer for exercise. They make decisions about *timing* and what are the highest priorities for discussion at that stage of treatment. This is negated with *opt-out* systems.	*“When you meet new people, they've usually got a hell of a lot going on. And is that the right time to talk about exercise?”* (Int-17)
	Other personal attributes (E)	Healthcare providers are committed, passionate and do more than is typically expected of their role (particularly evident of exercise delivery staff).	*“Everyone’s got the same passion for what we’re doing and that goes along way.”* (Int-7)
Process	Champion (E)	A champion exists who is influential with executives, peers and their direct reports. They use this influence to advocate for increased resources and funding. Champions also build other advocates (i.e., patients and peers) so there is a sense of *unity* in messaging around exercise EBIs and cancer.	*“My line manager is the best support champion of this program and has been the driver for this expanding over the years.”* (Int-2)
	Reflecting and evaluating (E)	Procedures are established to monitor and evaluate the implementation process, albeit this is not conducted in a systematic way. Sites use the information as needed to create the story they need to tell at that time.	*“So, when you start to see those patterns where either your activities are going up, wait lists are blowing out, a certain type of service is required because it's getting requested, etc. That's generally the driver behind pulling that data and doing a business case.”* (Int-16)

*B* Barrier, *COSA* Clinical Oncology Society Australia, *DR* document review, *EBI* evidence-based intervention, *EMR* electronic medical record, *E* enabler, *NA* not applicable

Sixteen determinants were identified that differed across sites. This included differences where some sites identified a determinant as a barrier while others recognised the same determinant as a facilitator of implementation (i.e., two sites viewed the lack of leadership engagement as a risk to the sustainability of exercise EBIs, while one site reported leaders were highly engaged and committed to EBI success). Five determinants listed in the CFIR were not identified at any locations. Supplementary file 3 summarises these determinants.

### Implementation strategies

Across case sites, the number of implementation strategies in use ranged from 36 to 44. We identified 22 implementation strategies that were consistent and in use across all case sites. Table 3 describes the consistent implementation strategies across sites, including strategies such as promoting adaptability, changing record-keeping systems and developing a quality monitoring system. Further, 24 implementation strategies were different across sites and 27 implementation strategies listed in ERIC were not adopted by any site (Supplementary file 4).

**Table 3:** Descriptive example of consistent implementation strategies identified across sites using the Expert Recommendations for Implementation Change taxonomy

Adapt and tailor to context	
Promote adaptability	Referrals are accepted through multiple mediums (i.e., email, phone, and formal referral forms). Multiple different types of exercise are available and offered to patients.
Change infrastructure	
Change record system	Systems (i.e., EMR) are changed, updated or developed to ensure that exercise is included and monitored, consistent with other cancer treatments.
Develop stakeholder interrelations	
Develop academic partnerships	Academic partnerships are used to initiate services. Academic partnerships continue to be developed to trial new interventions, expand existing services and support quality improvement efforts.
Identify and prepare champions	A champion exists who advocates for the exercise EBI. Typically this person is determined, respected in their field and able to transcend hierarchal structures to influence across the system (i.e., to influence delivery staff, organisational executives and policymakers).
Inform local opinion leaders	Influential people (i.e., specialists, nurses and (where position exists) care coordinators) are identified and engaged to promote exercise EBI. They promote the EBI via speaking roles at forums, testimonials in marketing materials, or because they have a *seat* at the executive table.
Involve executive boards	Status/progress reports that document the impact of exercise EBIs are developed and fed through to the executive level. The purpose of this is to secure buy-in for the EBI and support requests for increased resourcing.
Promote network weaving	Organisations facilitate opportunities for staff to network (i.e., social events, multi-disciplinary meetings) and build relationships across disciplines. These relationships are leveraged by staff to create efficiencies in workflows (i.e., *corridor conversations* to prompt referrals and dovetailing clinical appointments to create a seamless service for patients).
Engage consumers	
Increase demand	Consumer activism is fostered so that patients demand and act for the exercise EBI (i.e., involved in public presentation, drafting policy documents and leading petitions for the service).
Intervene with patients, consumers to enhance uptake and adherence	Multiple strategies are applied to enhance adherence to exercise EBIs (i.e., regular phone calls, maintaining an exercise diary, providing home exercise programs (i.e., using Physitrack), use of technology (i.e., tracking exercise via apps/pedometers) and organising social coffee catch-ups amongst patients).
Involve patients’ consumers and family members	Patients are engaged with implementation efforts via fundraising initiatives and raising the profile/value of the service (see *increase demand*).
Prepare patients and consumers to be active participants	A *soft-entry* approach is adopted across EBIs where the first contact offers a light-touch introduction to exercise. This aims to build the patients capacity and ownership over their involvement in the exercise EBI.
Use mass media	Organisations use mass media sources (i.e., social media, websites, print media) to raise awareness about the exercise EBI.
Provide interactive assistance	
	Strategies were not identified from this category that were consistent across sites.
Support clinicians	
Develop resource sharing agreements	Formal and informal relationships are established with community-based exercise services. These agreements are used to facilitate referral to other exercise programs in the area if the existing program is at capacity, or to offer an alternate exercise service if the core program does not meet consumer needs.
Facilitate relay of clinical data to providers	Clinical information about the patient’s engagement and progress through the exercise EBI is relayed to referral sources at regular intervals.
Train and educate stakeholders	
Conduct ongoing training	The workforce has access to regular ongoing training in cancer care (i.e., via journal club, professional development courses and one-off training courses in exercise oncology).
Develop educational materials	Organisations use a range of educational materials to support the delivery of exercise EBIs (i.e., exercise recommendations for managing fatigue, referral prioritisation forms, how to refer form, *scripts* that guide new staff in how to deliver a *typical* exercise EBI session).
Distribute educational materials	Dissemination of educational materials typically occurs via email blasts and regular internal communication channels (i.e., newsletters).
Use evaluative and iterative strategies	
Develop and implement tools for quality monitoring	Templates are developed that guide clinical and operational aspects of the EBI (i.e., initial assessment and re-assessment forms that guide subjective and objective assessments and established care plans, exercise programming forms, consumer attendance records, forms to track referral rates to programs).
Develop and organise a quality monitoring system	A system is developed (i.e., tracking through Excel or EMR) that pools the individual data collected through the quality monitoring tools to track the overall impact of the EBI. This information is used for corporate reporting, to develop business cases and to advocate for the EBI (see *involve executive boards*).
Obtain and use patient, consumer, and family feedback	PROs are collected typically via surveys or focus groups prior to a patient’s involvement in the program to inform the EBI content. Post-program satisfaction with the service is captured.
Use financial strategies	
Access new funding	Diverse funding sources are pursued to deliver exercise EBI. This includes funding from grants, donations, philanthropic organisations and fundraising efforts.
Place innovation on fee for service list	Delivery staffs are allied health professionals (AEPs/physiotherapists), with their services funded through the universal healthcare systems in Australia (Medicare) or activity-based funding (for in-hospital care).

*AEPs* Accredited exercise physiologists, *EBI* evidence-based intervention, *EMR* electronic medical record, *PROs* patient-reported outcomes

Across case sites, the highest proportion of implementation strategies fell within the ERIC category of engaging consumers. All five strategies from this category were identified and in use at every case site (i.e., increase demand, intervene with patients, consumers to enhance uptake and adherence, involve patients consumers and family members, prepare patients and consumers to be active participants, use mass media). In contrast, the lowest proportion of implementation strategies were categorised as use of financial strategies. Of the 11 strategies listed within this category, only two were identified and used by all case sites (i.e., access new funding, place innovation on a fee for service list).

### Implementation outcomes

#### Acceptability

Exercise services were reported as acceptable; however, the degree of acceptability varied. At one site, acceptability was directly linked to the individual characteristics of delivery staff. Colleagues respected and valued the AEP personally and the service they offered.

“And we have a lovely AEP, and I think thanks to (them), generally speaking, it’s actually very well received.” (Int-5)

In contrast, findings from another site suggested exercise EBIs were more acceptable when embedded with a multi-disciplinary program (i.e., survivorship program, prehabilitation program or multi-disciplinary youth service). However, there was a prevailing view that exercise alone was not a core cancer service.

“I don’t think it’s truly being endorsed at an organisational level, particularly within (service name). It’s often thought of as a top-on service that would be nice but is not really endorsed.” (Int-15)

#### Fidelity

Fidelity of implementation considered adherence to the EBI protocol, measured across two constructs:

#### Quality of service delivery

Two sites facilitated formal and informal learning opportunities and technical assistance to ensure staff maintained high-quality service delivery. One site required staff to undergo standardised training in exercise and cancer. The training program provided up to 12 hours of online content and was supplemented with approximately five hours of face-to-face training. Although fidelity of implementation was assumed through the provision of training and technical assistance, we did not identify evidence to suggest that the quality of the resulting service was monitored through formal mechanisms.

#### Dose/amount of program

EBI dose varied across sites from 15 to 39 contact hours, ranging from 8 to 26 weeks. It was only possible to determine fidelity for one element of the service at case site C. Two sites had completed evaluations to measure fidelity of implementation, and, in both cases, greater than 80% compliance with the EBI protocol was achieved. Further, one site had an ongoing process (via the electronic medical records (EMR)) for measuring adherence to the program protocol. This was documented for individual patients according to the criteria >75%, between 50 and 75% or <50% adherence. At the time of the case study assessment, however, it was not possible to aggregate this information to obtain a measure of fidelity due to data entry errors.

#### Penetration

The integration of the EBI within an organisation was measured at a service and sub-system level:

#### Service level

Service level penetration (calculated as eligible people who use the service/total number eligible) varied across sites from 17.2 to 80.6%. At the site with low penetration, the service model had recently been altered to improve penetration (i.e., more exercise EBI sessions added at different times to better meet demand). At the site with high penetration, an “opt-out” referral system for the exercise EBI operated (i.e., patients were automatically allocated to undergo a review with the AEP, for participation in exercise, as part of standardised intake assessments).

### Sub-system level

Sub-system level penetration was high across sites. All sites had established dedicated role/s for delivering exercise EBIs documented in position descriptions. Outcomes from the exercise EBI were captured in organisation-wide reporting alongside other key performance indicators. One site had recently participated in state-wide workforce planning to determine the long-term staffing requirements for people skilled and capable in exercise prescription. Two sites documented the exercise EBI within standard operating procedures (i.e., patient intake assessment procedures).

#### Sustainability

The extent to which the EBI was maintained and institutionalised within ongoing, stable operations at sites was evaluated according to ongoing program components, evolution over time, and a process in place to measure continued health benefits.

#### Program components and evolution over time

Program components that evolved over time were assessed primarily using the PSAT and secondly through interviews. The domains with the highest scores across sites were program adaption (*M*=5.8, SD ± 0.8), environmental support (*M*=5.3, SD ± 1.3) and program evaluation (*M*=5.0, SD ± 1.3). The domains with the lowest scores across sites were partnerships (*M*=4.2, SD ± 1.5), funding stability (*M*=4.3, SD ± 1.2), and strategic planning (*M*=4.6, SD ± 1.4). Within domain scores at individual case sites ranged from a low of 2.2 (partnerships, case site C (adult)) to a high of 6.3 (program adaption, case site B). Across sites, the highest mean sustainability score was achieved at case site B (*M*=5.4, SD ± 0.4), and the lowest sustainability score was achieved at case site C (adult) (*M*=3.7, SD ± 1.7). Table 4 summarises the PSAT findings at each site and across sites.

**Table 4:** Program Sustainability Assessment Tool results at each case site and across sites

Domain	Definition^a^	Case site A	Case site B	Case site C (youth)	Case site C (adult)	Cross-site scores
		Mean^b^ (SD)				
Environmental support	Having a supportive internal and external climate for your program	6.1 (0.6)	5.2 (0.6)	5.6 (0.3)	3.8 (2.2)	**5.3 (1.3)**
Funding stability	Establishing a consistent financial base for your program	5.2 (0.3)	3.4 (1.4)	4.9 (1.4)	3.8 (0.9)	**4.3 (1.2)**
Partnerships	Cultivating connections between your program and its stakeholders	5.1 (0.8)	5.2 (1.5)	5.0 (0.6)	2.2 (0.9)	**4.2 (1.5)**
Organizational capacity	Having the internal support and resources needed to effectively manage your program and its activities	4.8 (1.0)	5.4 (0.4)	5.6 (0.6)	3.7 (2.3)	**4.9 (1.2)**
Program evaluation	Assessing your program to inform planning and document results	4.6 (0.9)	6.2 (0.5)	5.4 (0.3)	4.2 (2.2)	**5.0 (1.3)**
Program adaptation	Taking actions that adapt your program to ensure its ongoing effectiveness	5.9 (0.7)	6.3 (0.6)	5.6 (0.3)	5.6 (1.5)	**5.8 (0.8)**
Communications	Strategic communication with stakeholders and the public about your program	5.3 (1.1)	5.7 (1.1)	4.2 (1.3)	3.5 (2.8)	**4.8 (1.6)**
Strategic planning	Using processes that guide your program’s direction, goals, and strategies	4.6 (0.7)	5.5 (0.1)	4.9 (0.8)	3.2 (2.6)	**4.6 (1.4)**
**Sustainability score**		**5.2 (0.7)**	**5.4 (0.4)**	**5.2 (0.3)**	**3.7 (1.7)**	

The bold is used to indicate the total for each section in the table

^a^Definitions as supplied

^b^Possible range; 1–7, with higher scores indicating areas of greater program strength

#### Continued health benefits

All sites had a process to monitor individual health outcomes of the EBI. The information on health outcomes was typically aggregated for research papers, business case development (i.e., to request an increase in resources), or for corporate reporting. Historical evaluations of exercise EBIs indicated that across sites and various health measures (i.e., improvement in function and quality of life, reduction in fatigue, anxiety, or depression, and meeting exercise guidelines), participation resulted in health benefits. For example, one site reported a mean 21 percentage point increase in people meeting aerobic exercise guidelines and a 24 percentage point increase in the number of people meeting resistance exercise guidelines after 12 months. A second site reported a mean reduction in cancer-related fatigue (21%), anxiety and depression (8–12%), and improvement across various quality of life domains (7–14%).

### Implementation Research Logic Model

**Fig. 1:**
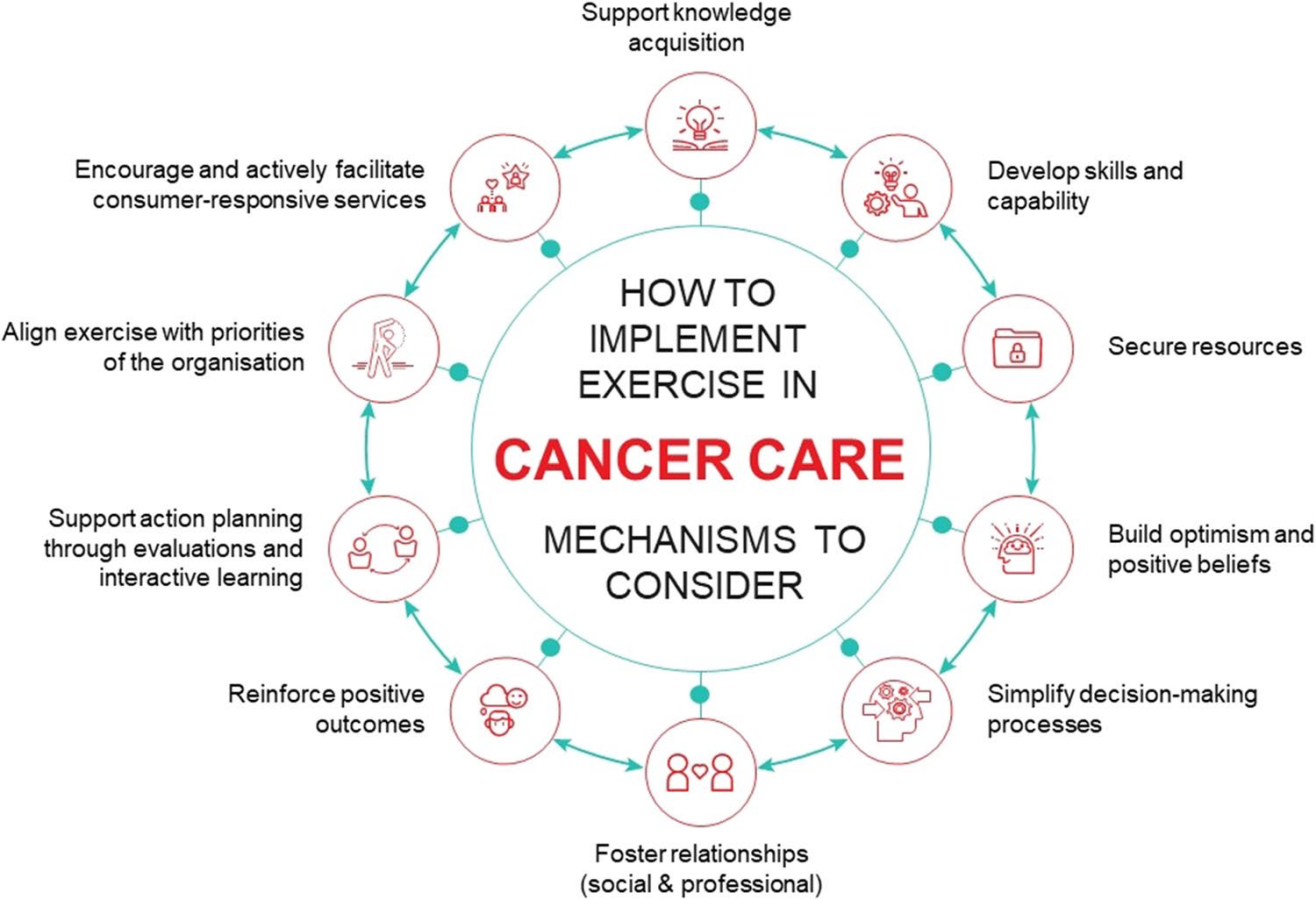
A summary of exercise implementation in cancer care

From the prioritised determinants, implementation strategies (and corresponding mechanisms), and implementation outcomes, a simplified IRLM was produced for each site (Supplementary file 5). By comparing and contrasting findings across the IRLMs, 11 common implementation pathways were developed that combined explain the implementation process (Fig. 1). A brief description of each pathway is provided and includes a rationale for how the proposed mechanism (italics) operates.

### Pathways contributing to the acceptability of the exercise EBIs

#### Develop knowledge about exercise via education and training

Sites applied strategies to train and educate stakeholders about exercise and cancer. This increased knowledge addressed a common barrier to implementation — a lack of confidence amongst staff to discuss exercise with patients. Education and training were targeted to develop both procedural and scientific knowledge. For example, hosting multi-disciplinary team meetings where program updates could be shared (procedural knowledge) and access to ongoing training so delivery staff maintained currency of knowledge (scientific knowledge).

#### Foster social and professional identity by developing relationships across the healthcare workforce

Strategies, including network weaving, were used to leverage social connections and develop professional role expectations and identity. These actions sought to augment the organisational dynamics and increase the strength and quality of networks and communication between healthcare providers. We hypothesise this motivated staff to align their behaviour with the expected functions of their role. Further, the qualitative data points suggested staff became more responsive to other clinicians’ needs through strengthened relationships.

“There is a lot of communication and that is a big strength of (site name), we have always been like a big family here. I sit in the same room as other oncologists and they can see the work I am doing and the meetings I am having with members of the team.” (Int-4)

#### Develop stakeholders’ skills and capability by adapting exercise EBIs to the changing context

Promoting the adaptability of EBI components developed stakeholders’ skills and capability in exercise. For patients, the type, dosage, and how exercise was delivered were all considered for adaption. The primary modification made for healthcare workers was to change referral methods. Adaptations increased skills and capability by facilitating mastery of the desired behaviour (i.e., patients can complete the prescribed exercise and healthcare workers make referrals to the service).

#### Build optimism and positive belief about the EBI by engaging stakeholders

Optimism about exercise EBIs was built through increasing the demand for the service. This was typically achieved by champions developing consumer activists who would advocate for the service. This resulted in changing stakeholders’ beliefs that motivated them to support the exercise EBI because it was perceived as the “right” option.

“I think the executive were very surprised that we had 700 signatures… so to see the amount of support we had.” (Int-3)

Acceptability was described differently across sites, which could be attributed to the degree to which differing implementation strategies were applied. For example, sites that invested more time in creating high-quality networks (or connections) experience higher levels of acceptability [55]. By contrast, organisational acceptance was low when the exercise EBI operated in isolation, as shown in the quote below:

“I guess that's kind of hard to tell…. so I think those that are directly involved with it think it's well received. But in terms of the wider scope, of outside of the department, I don't really know.” (Int-13)

### Pathway contributing to the fidelity of the exercise EBIs

#### Individuals engage in informal action planning via the provision of supportive, interactive assistance

Learning climate was a prioritised determinant across sites. Sites leveraged this climate by providing interactive assistance to ensure the quality of exercise EBIs. We theorise that the reciprocal nature of strategies such as facilitation and technical assistance encouraged an informal action planning method (i.e., changes in behaviour based on data). Protected time for problem-solving clinical and implementation issues existed across all sites.

#### Establish methods for ongoing evaluation and iteration that encourage change based on data

All sites tracked implementation via evaluation strategies, such as establishing quality monitoring systems or purposely re-examining implementation. Healthcare providers identified relevant measures of care, such as patient-reported outcomes or referral numbers, to monitor the service. The strategies encouraged planned changes to the service (action planning) based on acquiring targeted knowledge.

“We do track the effectiveness of what we're doing. So for example, if we get ten new inquiries each week and I only answer five of them, then obviously that’s a bit of an issue. At the end of every week, I’m tracking how many new inquiries we had. How many of those people have transitioned to being clients? And if they’ve declined –why? Is it too expensive? Do they live too far away? So trying to capture the reasons why people aren’t taking part and then we can use that data to address things.” (Int-6)

Arguably, the effectiveness of these strategies was enabled by the provision of resources that supported implementing with fidelity [56]. This included the provision of standardised training and templates to guide practice. Staff also had the autonomy to adapt and change procedures/processes based on the findings of ongoing learning.

### Pathways contributing to the penetration of exercise EBIs

#### Reinforce the expected outcomes of the EBI by supporting healthcare workers

A strategy applied across sites to support healthcare workers was relaying clinical data reinforcing the desired clinical behaviour. That is, for healthcare workers to act and make referrals to the exercise EBI. This strategy created a positive feedback loop because workers were exposed to the outcome of their actions which motivated the likely repetition of that behaviour in the future [57, 58].

#### Simplify decision-making processes associated with the EBI by creating the perception of a one-stop-shop

Easy access to information facilitated implementation across sites and was enabled by strategies that created the perception of a one-stop shop. Actions such as creating new clinical teams transformed exercise from an isolated intervention to a comprehensive program. This reduced the need for stakeholders to remember critical information about the exercise EBI and simplified the decision-making process by decreasing the cognitive load. In some cases, this extended to removing decision-making altogether through “opt-out” referral practices, as suggested in the quote below:

“So, the success of our programme has really been taking a much more macro approach to exercise, and embedding that within the core delivery of our program… (by contrast) if you have an add-on service then it's very, very hard for us to know who to refer to, and which patients we select to get it” (Int-15)

#### Create an aligned goal between the EBI and the organisation by capturing information that supports executive priorities

Organisations employed implementation strategies, such as involving executive boards that operated to align exercise EBIs with the priority goals of the organisation. That is, staffs were aware of the policy and funding levers within the outer setting and elevated the pulling effect of these determinants through to leaders [59]. They achieved this by ensuring leaders were provided with relevant information on the exercise EBI that aligned and contributed to organisational goals. For example, one report stated: “The governance of (site name) and its deliverables to both the (health department) and the Commonwealth Government remains the responsibility of (site name)…. On behalf of the (site name), we are pleased to submit our progress report to the (health department) which builds on the previous six month report” (DR–125)

These pathways supported both service-level (seven and eight) and sub-system level (nine) penetration. Service level penetration varied across sites (from low (17.2%) to high (80.6%)), which may be attributed to the scope of strategies taken to simplify decision-making (i.e., “opt-out” processes within the “one-stop-shop”). “Opt-out” referral meant penetration was not reliant on individual clinician behaviour (i.e., healthcare professionals discussing exercise with patients and then deciding (and acting) to make a referral).

### Pathways contributing to the sustainability of exercise EBIs

#### Grow and secure resources by accessing new funding and developing resourcing-sharing agreements

Available resources were a consistent barrier identified across sites. Organisations addressed this barrier by pursuing strategies, such as accessing new funding or creating resource-sharing agreements, which secured increased resources and provided more opportunities for the exercise EBI. Once extra resources were secured staff would work to hold the change and prevent the organisation from reverting back to the status quo.

“But we've grown (exercise) over time and been able to maintain that ring-fence. Because there’s been questions over time, should we convert it to physio,… we’ve been very mindful of the risk that it will be lost amongst all the other priorities.” (Int-16)

#### Drive consumer-responsive decision-making through leveraging interpersonal relationships coupled with action planning

Organisations implemented strategies, such as establishing consumer advisory committees that embedded patient needs within the service. These strategies brought together different stakeholder groups and created social change by influencing interpersonal relationships. Typically, opportunities to improve the service were pursued from these interactions or information gathered from consumers.

Securing resources and driving a consumer-responsive service are suggested to contribute to sustainability. However, we note that the actions described through preceding pathways (i.e., actions to increase acceptability, fidelity and penetration) also contributed to sustainability outcomes. To illustrate, skills and capability in exercise were developed through promoting adaptability, which contributed to acceptability. Program adaption was also identified as a strength contributing to sustainability (via the PSAT). Similarly, we hypothesised that evaluation strategies were necessary for enabling change based on data, contributing to implementation fidelity. Program evaluation was also a strength identified through the PSAT. Finally, the site that achieved high penetration levels spoke about an evolving service model consistent with our sustainability measures (i.e., evolution over time). Staff recognised the priorities of service users, and the organisation changed, and their model needed to change to remain relevant and acceptable to stakeholders, as shown in the quote below:

“So as our services evolve, we’ve evolved that model. And we piloted and tried some stuff, and then that hasn’t worked and we shifted on to other areas….so it’s a model that is kind of continuously evolving.” (Int-14)

**Fig. 2:**
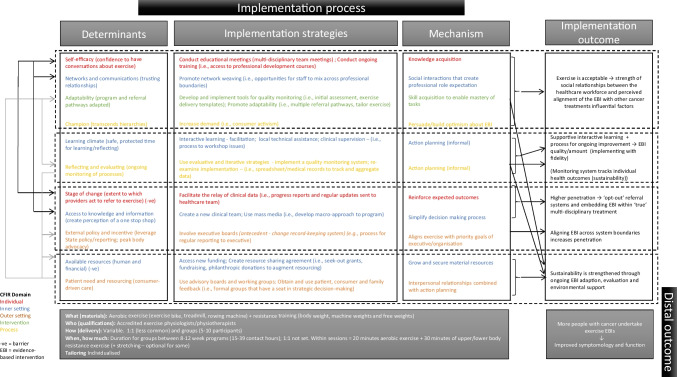
Implementation logic model for exercise evidence-based intervention in cancer care

Figure 2 combines these pathways within an implementation logic model to explain the implementation process of exercise EBIs in routine cancer care. The logic model groups each pathway according to its immediate implementation outcome. However, multiple arrows are added to the logic model to demonstrate the interrelationships between constructs and outcomes.

## Discussion

This is one of the first studies on exercise EBIs and cancer that systematically identifies determinants, implementation strategies and outcomes. Further, via multiple case study methodology, the program logic and relevant theoretical application, 11 causal pathways explaining the implementation process are proposed. These pathways represent potentially transferable elements that can be drawn upon to support future implementation efforts in exercise and cancer. In the following section, we discuss some of the key findings.

Our first study aim was to identify commonalities and differences in determinants, strategies, and outcomes. We commenced by considering each construct separately, viewed through the lens of the relevant framework, and consequently undertook a process to make sense of and unify these constructs through the IRLM [60, 61]. Davidoff (2019) describes this process as a mechanic needing to understand the different parts of a car before the vehicle can be repaired [60].

Consistent with other studies in exercise EBIs and cancer, multiple determinants that influence implementation were identified across all levels of implementation. Building on the existing knowledge in exercise EBIs and cancer we identified 11 determinants that, across different contexts and healthcare settings, were highly influential within the implementation process. While studies in exercise and cancer have applied the CFIR to guide study elements [32, 62–64], prioritising determinants is less common, despite being a way to identify those factors more likely to inform implementation success [65, 66]. Consistent with our findings, previous studies in exercise and cancer that have prioritised the determinants as most important for implementation success have identified: patient need and resourcing [31, 67, 68], available resources [67, 68], adaptability [31], reflecting and evaluating [31], and external policies and incentives [68]. More broadly, implementation scientists have identified patient need and resourcing (relative advantage and tension for change), as factors associated with implementation success across multiple studies [66] and available resources as a highly prominent determinant [69]. Our findings can be applied prospectively to focus attention in needs assessments that plan to implement exercise in cancer care. Prioritising determinants can help with selecting and matching implementation strategies.

Across sites over 30 implementation strategies were used to support implementation, with 22 strategies common in all sites. This figure is consistent with other implementation research that suggests organisations typically employ numerous strategies [70–73]. To our knowledge, of studies that use the ERIC taxonomy to document implementation strategies in exercise EBI and cancer, many report fewer than ten strategies [42, 74], with only one other study conducting comprehensive mapping [34]. Our process to identify strategies and then apply an inductive approach to develop the explanatory pathways helps address the identified gap between the number of strategies prospectively included in implementation trials and the actual number used when retrospectively identified [75]. We identified several plausible strategies within the individual pathways that can now be applied prospectively. Several methods have been trialled to support pragmatic documentation of implementation strategies in research and practice [70, 73, 76, 77]. It is also feasible for non-specialists to accurately identify strategies when supplied with a standardised list [78]. Future studies can build on our work to develop a knowledge bank of implementation strategies most helpful for integrating exercise into cancer care. This may also include concurrent reporting of implementation strategies in clinical trials and conducting hybrid effectiveness-implementation trials that provide crucial information about how to implement, alongside understanding the clinical impacts of the EBI [76, 79].

The third area where we sought to identify commonalities and differences across sites was by evaluating implementation outcomes. Evaluations of implementation outcomes help to define implementation success. However, it could be argued that some of the exercise EBIs we evaluated had limited success, noting some sites exhibited low penetration rates, low PSAT scores, and differing levels of acceptability. It is probable that implementation outcomes need not be compartmentalised as successfully achieved or not but viewed as to what extent the outcome has been achieved [80]. Staff continually flexed and evolved in response to their environment and made changes to the EBI in response to an implementation outcome. To illustrate, delivery staff often changed EBI components and made decisions during a consultation about the most critical element to deliver on that day, which likely impacts implementation fidelity. Staff would also self-organise to pursue new funding opportunities as they arose, potentially influencing perceptions of sustainability. By contrast, in response to low penetration outcomes, staff changed the service delivery model to better integrate exercise EBIs into existing clinical workflows.

Notwithstanding the fluidity of implementation outcomes two key findings are highlighted from our work. First, consistent with an update to the CFIR [81], we identified a relationship between acceptability and other outcomes, suggesting acceptability is an antecedent that may predict actual implementation outcomes. The site where staff reported lower awareness of the exercise EBI also achieved low penetration (service level) and the lowest scores on the PSAT (sustainability). These findings confer with recommendations to measure acceptability early in the implementation process to understand whether the organisational conditions are suitable for implementation [20, 82]. Second, creating an “opt-out” referral system was associated with higher penetration. This is consistent with findings that indicate creating default options that direct healthcare providers down a path of least resistance increases referrals (in cardiac rehabilitation) [83, 84]. In our study, sites developed an “opt-out” system when clear eligibility criteria were established for EBIs and resourcing matched the anticipated demand for the service. There is a need to consider how “opt-out” referral systems may operate in exercise and cancer to increase penetration. Kennedy and colleagues have recently described their efforts to create an “opt-out” system in exercise and cancer by developing an integrated workflow, which resulted in a three-fold increase in program reach [85]. This could work in tandem with recent work to develop exercise oncology clinical pathways and decision-support tools to increase the uptake of exercise EBIs in cancer care [8, 86, 87].

The second main aim of this study was to develop an explanatory causal pathway for how implementation occurred. This work provides important insights into the transferable elements that can be applied in future implementation efforts. Critically, these pathways exhibit interrelatedness, rather than being isolated, linear, cause-and-effect processes. Recognising this complexity and identifying the function or mechanism theorised to produce change can provide guidance when considering the transferability of findings. This encourages reflection about how strategies lead to behaviour change rather than just identifying what the strategy is [57]. To illustrate, based on findings from our case studies, a range of evaluation and iterative implementation strategies were applied. However, we suggest the mechanism of change was a form of action planning. Similarly, training and education opportunities varied; however, the mechanism was to increase knowledge. The practical implications of these findings are that multiple strategies might be suitable to perform a function, however, drawing together a bundle based on their mechanisms and ability to directly influence determinants may help focus efforts. The disciplines understanding of mechanisms is still in a formative stage, with work underway to identify common strategies/mechanism relationships by some researchers [88, 89]. To our knowledge, only one other study in exercise and cancer has sought to identity mechanisms [42]. Similar to our findings, Kennedy and colleagues identified strategies from across ERIC categories that sought to increase knowledge, secure resourcing (financing) and improve intra-organisational communications. Our approach used relevant frameworks and multiple case study methods coupled with program logic to propose mechanisms. This may be considered an early stage of developing transferable elements [90]. These relationships require empirical testing, with refinement expected from those outcomes. These pathways provide direction on the suite of actions needed to support successful implementation in exercise and cancer. They provide a starting point for conversations and planning between stakeholders seeking to implement exercise EBIs in cancer care.

## Limitations

This study used a novel and comprehensive approach to develop a synthesised logic model of the implementation process. Nevertheless, some limitations need to be addressed. Lewis and colleagues recommend identifying mediators and pre-conditions when developing causal pathways [22]. Sales and colleagues suggest mechanisms of determinants, in addition to mechanisms of implementation strategies, should also be identified when using the IRLM [91]. Further, the IRLM does not specify a framework to explicate mechanisms. Although we considered existing literature, we did not explicitly apply a framework to identify mechanisms. Implementation scientists have suggested this is possible with the ERIC and behaviour change technique (BCT) taxonomy (which underpins the behaviour change wheel (BCW)) [92]. Through secondary analysis of the case study database, it would be possible to deconstruct individual pathways further to identify mechanisms of determinants, moderators and pre-conditions. Additionally, a comparative analysis could be undertaken to match the mechanism with the source of behaviour on the BCW (i.e., capability, opportunity, motivation), however, was outside the scope of this study.

COVID-19 interrupted our planned data collection meaning fewer hours of onsite observations were conducted at one case site. The study also selected participants who had a working knowledge of the exercise EBI to understand their views and experiences. Seeking out a broader range of stakeholders, including those who have no knowledge of the service may elicit useful insights. There is also a need to test and replicate our approach across more sites given the formative nature of our work in exercise and cancer.

## Conclusion

In summary, we identified commonalities in determinants and strategies (including mechanisms) that facilitated the development of potentially transferable explanatory causal pathways for exercise EBIs in cancer care using a multiple case study approach. The pathways we identified were interrelated and dependent upon each other to produce the resulting outcomes. By identifying mechanisms of change, we demonstrate that multiple strategies are needed for successful implementation as they may contribute to change in different ways and lead to different outcomes. Future studies can build on this work by empirically testing various elements of the hypothesised causal pathways and applying our findings prospectively to develop implementation plans. This is one of the first studies in exercise and cancer that, across multiple sites, systematically applies and then combines multiple implementation science frameworks to explain the “how and why” of implementation. These findings can support efforts to scale exercise EBIs as a standard component of cancer care.

## Supplementary information

ESM 1

ESM 2

ESM 3

ESM 4

ESM 5

## Data Availability

The data that support the findings of this study are available on request from the corresponding author [LC]. The data are not publicly available due to containing information that could compromise research participant privacy.
