# Supplementary material for: “Now is the time for institutions to be investing in growing exercise programs as part of standard of care”: a multiple case study examining the implementation of exercise oncology interventions
Source: Support Care Cancer. 2023 Jun 26;31(7):422. doi: 10.1007/s00520-023-07844-x (PMC10293395; doi:10.1007/s00520-023-07844-x)
Supplement: Supplementary file 1 — ESM 1 [file 520_2023_7844_MOESM1_ESM.docx]

**Supplementary file 1: Description of exercise evidence-based interventions implemented at case sites**

|  | **Case Site A** | **Case Site B** | **Case Site C (youth)** | **Case Site C (adult)** |
| --- | --- | --- | --- | --- |
| **What (equipment)** | Aerobic exercise (treadmill, seated rowing machine, exercise bike)  Resistance training (free weights, machine weights, resistance bands) | Aerobic exercise (treadmill, exercise bike)  Resistance training (free weights, machine weights) | Aerobic exercise (exercise bike)  Resistance training (body weight, resistance bands, free weights) | Aerobic exercise (exercise bike)  Resistance training (body weight, resistance bands, free weights) |
| **Who (qualifications)** | AEP | AEP + completion of cancer-specific training course (12 hours online training and 4–5-hour face-to-face workshop) | AEP | AEP and physiotherapists |
| **How (delivery)** | Group program (1 AEP per 5-6 participants) | Group program (1 AEP per 5-10 participants) | 1:1 (during the active treatment phase)  Group programs (within *optional* survivorship program) | Patient choice  1:1 session, home-based, or referral to a community provider  Group sessions run 3x/week |
| **When, how much (dosage)** | 12-week program (15 contact hours)  1x20 minute introduction + 1x1 hour initial assessment  1x1hr/week for 12 weeks  1x1 hour final assessment  1x20 minute review at 4 and 6 months  Sessions include:  20 minutes aerobic exercise  30 minutes of resistance exercise (6-8 upper and lower body exercises)  10 minutes (group) stretching  Nb: 7 different times for group classes are offered between Tuesday and Friday | 12-week program (39 contact hours):  2x1 hour initial assessment  3x1 hour session per week (for 12 weeks)  1x1 hour final assessment  Sessions include:  20 minutes of aerobic exercise 30 minutes of resistance (6 upper and lower body exercises (2-4 sets x 6-12 repetitions))  Nb: people select 1 of 5 sites to attend and are provided with a 3-month gym membership included in program cost | 1:1 program  Duration not set  1x30 minute initial assessment  Session duration varies between 20 mins - 1 hour  Sessions include:  20 minutes aerobic exercise 30 minutes of resistance exercise (8 upper and lower body exercises (2 sets x 10-12 repetitions)  10 minutes stretching  *(preference is given to aerobic exercise when time is limited)*  Group program  8-week program (16.5 contact hours)  1x30 minute initial assessment  2x1 hour sessions per week  (1 hour exercise (content as per 1:1) + 1 hour education) | Duration not set  1x30 minute initial assessment  Session duration varies between 20 mins - 1 hour duration (onsite)  Re-assessment as required  Onsite sessions include:  20 minutes aerobic exercise  30 minutes (8 upper and lower body resistance exercise (2 sets 10-12 repetitions)  10 minutes stretching  *(preference is given to aerobic exercise when time is limited)* |
| **Tailoring** | Exercise EBIs are individualised.  Adherence prompts (Physitrack* exercise sheets, walking checklist, use of fitness trackers or pedometers and exercise diaries) | Exercise EBIs are individualised.  Adherence prompts (home program promoting 3x30 minute aerobic exercise per week) | Exercise EBIs are individualised.  Adherence prompts for groups (custom-made app to track exercise, podcasts, videos, supports maintenance, fatigue and physical activity diaries) | Exercise EBIs are individualised.  Adherence prompts (Simpleset* exercise program, education, fatigue and physical activity diaries) |
| ***Clinical assessment template*** | Template guiding initial assessment.  Information captured:  1) referral source  2) cancer history  3) objective assessment (grip strength, 30-second sit-stand)  4) subjective measures including symptomology and patient goals, barriers and enablers to exercise, FACT-G  5) treatment recommendations | Template guiding initial assessment.  Information captured:  1) pre-program questionnaire  2) prompt to ‘develop rapport’  3) medical history  4) patient goals  5) objective assessment (height, weight, blood pressure, heart rate, 6-minute walk test, 30-second sit/stand)  6) treatment recommendations | Triage via HEEADSSS assessment framework.  Initial assessment template (refer to Case site C (adult) | Template guiding initial assessment.  Information captured:  1) medical history including cancer and lifestyle factors  2) assessment performance status (AKPS score and self-reported physical activity levels)  3) precautions for exercise  4) physical examination (height, weight, mobility, independence with transferring, resting heart rate, oxygen saturation and blood pressure), functional tests (6-minute walk test, grip strength, sit-to-stand test)  5) patient goals and objectives |
| ***Exercise session template*** | Template guides exercise session.  Information captured:  1) name of exercise  2) weights, repetitions and sets for each resistance exercise  3) adherence and fatigue during session  4) prompts review of program every 2 weeks | Template guides exercise session.  Information captured:  1) session number  2) name of exercise  3) weight, repetitions and sets for each resistance exercise  3) intensity of aerobic exercise  4) adherence and fatigue during session  5) prompts review of program every 2 weeks | Template guides exercise session.  Information captured:  1) exercise type  2) intensity of aerobic exercise  3) heart rate/oxygen saturation  4) template captures up to 8 weeks of programming  Program adherence is captured through EMR and not on exercise template | Template guides exercise session.  Information captured:  1) aerobic and resistance exercise prescribed  2) intensity, frequency, duration, repetitions, and sets.  Adherence to program captured after each session through EMR |
| AEP = Accredited Exercise Physiologist, AKPS = Australia-modified Karnofsky Performance Status, EMR = Electronic Medical Record, HEEADSSS = acronym for a comprehensive psychosocial assessment tool, FACT-G = Functional Assessment of Cancer Therapy – General, * = Physitrack and Simpleset are exercise software packages | | | | |
