# Supplementary material for: “Now is the time for institutions to be investing in growing exercise programs as part of standard of care”: a multiple case study examining the implementation of exercise oncology interventions
Source: Support Care Cancer. 2023 Jun 26;31(7):422. doi: 10.1007/s00520-023-07844-x (PMC10293395; doi:10.1007/s00520-023-07844-x)
Supplement: Supplementary file 2 — ESM 2 [file 520_2023_7844_MOESM2_ESM.docx]

**Supplementary file 2: Study aims, conceptualisation with operational measurement**

| **Study aim** | **Conceptualisation** | **Operationalised measurement** |
| --- | --- | --- |
| **Identify the commonalities and differences in determinants, implementation strategies and implementation outcomes (acceptability, fidelity, penetration, and sustainability) across exercise oncology services** | | |
| *Identify determinants* | CFIR | The CFIR framework lists 39 determinants across 5 domains and has an accompanying interview tool that was used to develop questions for the semi-structured interview guide that elucidated the determinants influencing implementation. |
| *Identify implementation strategies* | ERIC | The ERIC taxonomy lists 73 implementation strategies (including Additional file 6) and provided the description of implementation strategies identified and categorised through the study. |
| *Identify implementation outcomes* | IOF | The IOF is an evaluation framework that lists 8 proximal outcomes for measurement in implementation studies, of which 4 were evaluated in this study (see below). |
|  | *Acceptability - The perception among implementation stakeholders that a given treatment, service, practice, or innovation is agreeable, palatable, or satisfactory* | Interview question - How do you think the exercise intervention is perceived within your organisation? |
|  | *Fidelity - The degree to which an intervention was implemented as it was prescribed in the original protocol or as it was intended by the program developers* | Adherence to the program protocol according to amount and quality  *Quality of program delivery*  Training to support quality delivery  *Amount of program delivered/received*  # Sessions/duration per week  # Total program delivered (delivered)  # Attendance rates (received) |
|  | *Penetration - The integration of a practice within a service setting and its subsystems* | *Integration in the service system*  # Eligible people who use the service/total number eligible  *Integration in the subsystems*  # Documented position description where the intervention is defined in a role  # Budget that confirms funding  # Strategic planning that includes reference to the intervention |
|  | *Sustainability - The extent to which a newly implemented treatment is maintained or institutionalized within a service setting ongoing, stable operations* | Sustainability is measured according to continued EBI components, the evolution of the EBI over time and process in place to assure continued health benefits  *Continued program components*  PSAT + Interview question: Are there any parts of the intervention that should not be changed?  *Evolution over time*  PSAT + Interview questions: Has what was implemented changed over time? What kinds of changes or alterations have been made to the intervention so it continues to work effectively in your setting?  *Continued health benefit*  Interview question: What type of objective health measures are taken before, during or after the program to monitor the patient’s response to the exercise? |
| **Develop an explanatory causal pathway for the implementation process from the common elements that exist across services** | | |
| *Explain implementation* | IRLM | A simplified IRLM was developed for each site and then the common elements identified across IRLMs were extracted, synthesised and reproduced in a single logic model |
| CFIR = Consolidated Framework for Implementation Research, EBI = Evidence-based Intervention, ERIC = Expert Recommendations for Implementing Change, IRLM = Implementation Research Logic Model, IOF = Implementation Outcomes Framework, PSAT = Program Sustainability Assessment Tool | | |
