# Supplementary material for: “Now is the time for institutions to be investing in growing exercise programs as part of standard of care”: a multiple case study examining the implementation of exercise oncology interventions
Source: Support Care Cancer. 2023 Jun 26;31(7):422. doi: 10.1007/s00520-023-07844-x (PMC10293395; doi:10.1007/s00520-023-07844-x)
Supplement: Supplementary file 3 — ESM 3 [file 520_2023_7844_MOESM3_ESM.docx]

**Supplementary file 3: Descriptive example of determinants that were different across sites, identified using the Consolidated Framework for Implementation Research**

| **Domain** | **Construct** | **Description of determinant** | **Example** |
| --- | --- | --- | --- |
| Intervention | Intervention source | NA |  |
|  | Complexity | Two sites had implemented a centralised and streamlined referral process (i.e., through electronic medical record and a bespoke IT system) that sought to address complexity. One site identified the referral processes to exercise as *more work*. | *“Soon as that referral comes, it comes in through (an IT system), which centralizes everything into a single point contact for those guys.” (Int-09)*  *“But because it's a change to routine practice because it's an extra element now added into a consultation.” (Int-02)* |
|  | Design quality and packaging | One site had created a consistent brand and image for marketing the exercise EBI, which added to its perceived excellence. By contrast, the lack of packaging and bundling of the program for use was viewed as a barrier at another site. | *“And then obviously people were looking at the website and going this program looks great.” (Int-06)* |
|  | Cost | One site had costed the EBI ($1000.00AUD) and embedded this knowledge within how the service was implemented/funded and delivered. | *“So the cost is a thousand dollars. We try and fundraise to subsidise that cost so that we can provide access to as many patients as possible.” (Int-09)* |
| Outer setting | Peer pressure | Two sites viewed their organisation as a *world leader* that set an agenda for other organisations. One site saw exercise EBIs as the *norm*, resulting in rapid implementation across the healthcare setting. | *“Everyone we meet every day knows about (organisational name).” (Int-04)* |
| Inner setting | Structural characteristics | Exercise EBIs were limited by the size of the allocated room and the equipment available. Further, two sites reported the organisational reporting line for exercise EBI was wrong and created barriers to implementation. By contrast, one site planned and selected sites based on specific criteria (i.e., parking, close to public transport, coffee shop and a range of equipment). | “*We have a dedicated gym space. Again, probably would like different pieces of equipment. Some of its more geared to sort of acute rehab, which don’t really get seen, so... in hindsight, you wouldn't need that space, whereas capacity for exercise has really grown in that space and demand, you would probably change how you allocate space.” (Int-11)* |
|  | Culture | Staff across sites expressed different opinions about organisational culture. Some expressed the view that culture was *poor*. Other sites saw their culture as supportive and collegiate. | “*I would divide it up, I think the (immediate team) are a fantastic team and we work really well together. I think we are very supportive of each other, and it is probably what keeps me coming to work each day. Bigger than that is the (oncology department culture) and that is less supportive. Then outside of that is just working in a (organisation name), and at the moment that is just cuts going left right and centre, and (that is) not supportive.” (Int-01)* |
|  | Tension for change | Tension for change was not high enough to force change in behaviour/practice that would facilitate integration of exercise in routine practice. | *“We grow as big as the organisation wants us to grow. So there's like gatekeeper issues there. And if we're not generating a lot of revenue for the organisation, there's limited incentive to grow that service.” (Int-14)* |
|  | Relative priority | Two sites reported that in busy, time-limited consultations healthcare providers make decisions about what is the highest priority for discussion at that time. | *“The clinical imperative at the time is perhaps to look at different things. It is not seen as ... clinical important at a particular time when the doctor may be seeing the patient.” (Int-05)* |
|  | Organisational incentives and rewards | At one site the service had recently won a prestigious award, and this was seen as something that could be leveraged by the exercise EBI to support implementation. | *“We have been lucky that it has picked up some accolades along the way.” (Int-01)* |
|  | Goals and feedback | NA |  |
|  | Readiness for implementation | At one site, staff expressed a view that the exercise EBI was unplanned and disorganised. | “*I think it's just very haphazard and I think that's more to do with what we are as (organisation name) and the location of our patients. I think that plays a huge role in terms of the service that we deliver and what patients can access.” (Int-13)* |
|  | Leadership engagement | Two sites observed that lack of supportive strategic leadership was a risk to the sustainability of exercise EBI (i.e., because leaders didn’t understand and value the service they were less likely to see a need to resource it). By contrast one site viewed their leaders as highly committed and engaged in ensuring the success of the exercise EBI | *“Sometimes I think the decisions are made by not necessarily taking a macro approach...but rather it becomes so discipline specific that when there's funds available, it's sometimes who's got the loudest voice? Who's got the greater numbers?.. So I think that having, sometimes stronger executive leadership…clearer, overarching organisational strategy and goals, and ensuring that rather than just being in a document, that they translate down to our levels and that it should be a two-way process.” (Int-14)* |
| Individual | Knowledge and beliefs about the intervention | Different views about exercise EBIs existed across organisations. Some viewed it as beneficial, whilst others had established ideas about which patients should be referred and would benefit. | “*It could also be the perception that different groups are more appropriate for exercise. It maybe be and I don’t know this, but a younger woman with breast cancer is more likely to be referred than an older man with prostate cancer and other multiple co-morbidities and things like that.” (Int-05)* |
|  | Individual identification with the organisation | At one site staff demonstrated different levels of commitment to the organisation, which may have contributed to some sites being more successful than others. At other sites, staff expressed a view of feeling fortunate to work at the sites given their reputation as *world-leader*. | *“No, it doesn't worry me. It doesn't worry me because I know I've got the safety net of another job.” (Int-07)* |
| Process | Planning | One site undertook 12 months of planning prior to the launch. Other sites did not undertake systematic planning with the exercise EBIs growing opportunistically. | *“Yes and no. I guess in my mind this would go beyond the (research) study… but did I sit down 10 years ago and say as of now I want 1, 2 and 3 - no. But it was always I want more time for AEP, I want to be able to offer classes (and) increase the classes. Most of this has been in my head. At different times, yes we have had to write and say what we are planning and so I would have given some stats.” (Int-03)* |
|  | Engaging | Differential views existed across sites about who needed to be engaged. One site viewed the oncology team as most important because they gave patients *permission* to exercise. Other sites viewed middle management (who develop business cases) and nurses (who coordinate referrals) as the most important stakeholders | *“(The oncology team) they're the ones that push it in terms of encouraging their patients to do exercise, to engage in exercise and to give them permission to actually exercise.” (Int-09)* |
|  | External change agents | NA |  |
|  | Opinion leader | NA |  |
|  | Formally appointed internal implementation leader | NA |  |
|  | Executing | One site had established KPIs to track implementation progress. However, had not reviewed progress due to the impacts of COVID-19. | *“So I suppose we haven't had to measure ourselves against (KPIs) because we know that we just haven't done anything.” (Int-09)* |
| AEP = Accredited Exercise Physiologist, AUD = Australian Dollar, EBI = Evidence-based Intervention, IT = Information Technology, KPIs = Key Performance Indicators | | | |
