# Supplementary material for: “Now is the time for institutions to be investing in growing exercise programs as part of standard of care”: a multiple case study examining the implementation of exercise oncology interventions
Source: Support Care Cancer. 2023 Jun 26;31(7):422. doi: 10.1007/s00520-023-07844-x (PMC10293395; doi:10.1007/s00520-023-07844-x)
Supplement: Supplementary file 4 — ESM 4 [file 520_2023_7844_MOESM4_ESM.docx]

**Supplementary file 4: Implementation strategies that differed or were not in use across sites, identified using the Expert Recommendations for Implementation Change taxonomy**

| **ERIC strategy** | **Descriptive example** |
| --- | --- |
| **Adapt and tailor to context** | |
| Tailor strategies | Two sites tailored referral processes. One site trialled multiple systems to determine the most effective approach (i.e., case management model, triaging, all staff participating in the initial assessments and a hybrid model of triage based on physical, education, social and health needs). |
| Use data experts | One site had established a dedicated research role to support evidence-informed care. The position assisted staff in establishing outcome measures for the services and undertaking rapid reviews for use in business cases. |
| Use data warehousing techniques | NA |
| **Change infrastructure** | |
| Change physical structure or equipment | Two sites had created new purpose-built spaces for their patients (i.e., a new bigger gym space and a *chill-out* space for patients that felt *non-medicalised*). |
| Change service sites | One site operated a *hub and spoke* model that ensured exercise sites were accessible for patients. A second site had moved to a purpose-built facility which allowed for more dedicated exercise space. |
| Start a dissemination organisation | One site established a not-for-profit organisation with the sole purpose of providing evidence-based exercise for people with cancer. |
| Change accreditation or membership requirements | NA |
| Change liability laws | NA |
| Create or change credentialing and or licensure standards | NA |
| Mandate change | NA |
| **Develop stakeholder interrelations** | |
| Build a coalition | One site engaged organisations and individuals with specific expertise to provide in-kind financial, legal and regulatory support. This assisted the organisation to meet its corporate requirements. |
| Use advisory boards and working groups | Two sites established advisory groups, with representation from consumers, to support implementation efforts. At one site the advisory group focused on supporting organisational governance. At the second site, the advisory group had a specific policy development role. |
| Visit other sites | Two sites conducted site visits to other organisations delivering exercise oncology services to support initial implementation efforts. |
| Capture and share local knowledge | NA |
| Conduct local consensus decision | NA |
| Develop an implementation glossary | NA |
| Identify early adopter | NA |
| Model and simulate change | NA |
| Obtain formal commitments | NA |
| Organise clinical implementation team meetings | NA |
| Recruit, designate and train for leadership | NA |
| Use and implementation advisor | NA |
| **Engage consumers** | |
| **Provide interactive assistance** | |
| Centralise technical assistance | One site had established a centralised IT system which was described as the control centre for the service. The centralised system was managed by a coordinator and has several functions, including: streamlining referrals, payments and reporting, coordinating the care pathway and providing expert advice to other clinicians including education and evaluation support. |
| Facilitation | One site through ongoing involvement in research trials, scheduled regular team meetings to workshop implementation issues associated with the research trials. The outcomes of these meetings were then applied more broadly to the exercise EBIs delivered at the site to optimise services. |
| Provide clinical supervision | One site had established a clinical supervision model where all healthcare providers were provided with fortnightly and then monthly sessions with a more senior colleague. This model provided clinical and non-clinical support for the workforce. |
| Provide local technical assistance | At one site a dedicated role existed that supported EBI delivery staff with operational issues (i.e., reporting issues, how to use the IT system and processing payments and guidance on clinical issues). This was an ad hoc, informal system of support. |
| **Support clinicians** | |
| Create new clinical teams | Two sites had created multi-disciplinary teams that provided a wrap-around service for patients. Members of the multi-disciplinary team were upskilled in their colleague’s professional expertise. |
| Remind clinicians | One site had established a reminder system for the exercise EBI. This included: stickers placed on medical files prompting referral to exercise and adding the COSA exercise guidelines to the footer of the organisation’s letterhead. Two sites used informal reminder systems (i.e., arranging coffee catchups with referral sources and leaving flyers about the service in common areas). |
| Revise professional roles | One site had revised roles over time to grow and enhance the exercise services. It commenced with converting an allied health assistant role to an exercise physiology role. This position was then *ring-fenced* to avoid it being taken away through the growth/retraction of organisational budgets. The initial role has slowly been added to, with more hours and number of AEPs. The original AEP has progressed to a higher level in the organisation, increasing their responsibilities and decision-making capacity. |
| **Train and educate stakeholders** |  |
| Conduct educational meetings | Two sites conducted multi-disciplinary team meetings where staff could provide colleagues with updates on discipline-specific initiatives, such as progress on exercise EBI. |
| Create a learning collaborative | At one site the health district established a learning collaborative for AEP staff working at different sites. Separately, a collaborative that included multiple different healthcare disciplines was also established to support shared learning. |
| Provide ongoing consultation | At one site a secondary consultation service operated that provided advice to other clinicians delivering cancer services. This service included medical, nursing and allied health services (including exercise). |
| Shadow other experts | NA |
| Work with educational institutions | One organisation created an educational coordinator role. This role worked with a local university to deliver post-graduate courses. The role also worked with other organisations to develop internal and external training opportunities that built general workforce capacity. |
| Conduct educational outreach visit | NA |
| Make training dynamic | NA |
| Use train the trainer strategies | NA |
| **Use evaluative and iterative strategies** | |
| Assess for readiness and identify barriers and facilitators | Prior to establishing the service one site spent 12 months planning, identifying potential barriers and engaging with stakeholders to improve the likelihood of implementation success. |
| Audit and provide feedback | At one site staff were required to undertake an annual quality improvement project of their choice. The AEP was conducting a project to monitor the current triage process for the exercise service. The aim of this project was to improve the *right referral at the right time*, with the results planned for feedback to the triaging workforce. |
| Conduct cyclic small tests of change | At one site home exercise resources were trialled with a small group of patients before implementing widely in the service. |
| Stage implementation and scale-up | Two sites built the exercise EBI services over time (i.e., adding extra sessions/times and delivering exercise for different cancer streams). As the exercise EBI is expanded, efforts are then directed towards ‘locking’ the change into the system. |
| Purposely re-examine the implementation | One site only set exercise timetables for a term at a time. At the end of each term they re-assessed attendance rates to ensure class days/times were suitable for participants. |
| Conduct a local needs assessment | NA |
| Develop a formal implementation blueprint | NA |
| **Use financial strategies** | |
| Alter incentives allowance structures | NA |
| Alter patient, consumer fees | NA |
| Develop disincentives | NA |
| Fund and contract for the clinical innovation | NA |
| Make billing easier | NA |
| Use capitated payments | NA |
| Other payment schemes | NA |
| AEP = Accredited Exercise Physiologists, COSA = Clinical Oncology Society Australia, EBI = Evidence-based Intervention, IT = Information Technology, NA = Not Applicable | |
