# Supplementary material for: “Now is the time for institutions to be investing in growing exercise programs as part of standard of care”: a multiple case study examining the implementation of exercise oncology interventions
Source: Support Care Cancer. 2023 Jun 26;31(7):422. doi: 10.1007/s00520-023-07844-x (PMC10293395; doi:10.1007/s00520-023-07844-x)
Supplement: Supplementary file 5 — ESM 5 [file 520_2023_7844_MOESM5_ESM.docx]

**Supplementary file 5: Simplified implementation logic model for Case Site A, B and C**

| **Case Site A** | | | | |
| --- | --- | --- | --- | --- |
| **CFIR construct** | **Summary of construct extracted from framework matrix** | **Implementation strategy (ERIC)** | **Mechanism** | **Implementation outcomes** |
| **Individual (E)** | **Self-efficacy (healthcare providers have the confidence to discuss exercise)** | **Conduct education meetings (multi-disciplinary team meetings)**  **Conduct ongoing training (access to professional development courses)** | **Knowledge acquisition** | **Acceptability (provider)** |
| **Individual (B)** | **Stage of change (some healthcare providers act to refer, others don’t)** | **Facilitate relay of clinical data to providers (patient progress sent to referral source at regular intervals through the program)** | **Reinforces expected (positive) outcomes** | **Penetration (service level)** |
| Inner setting (B) | Culture (poor culture across the broader organisation) | Promote network weaving (create opportunities for staff to mix across professional boundaries) | Social interaction | Acceptability (provider) |
| Inner setting (E) | Implementation climate (within the small team the program is agile, which allows it to adapt and respond despite austerity) | Create new clinical teams (multi-disciplinary team) | Professional role identity and responsibilities | Acceptability (provider)  ↓  Penetration (service system) |
| **Inner setting (E)** | ***Learning climate (ongoing learning permeates the organisation)*** | ***Facilitation (protected time to workshop implementation issues)*** | ***Action planning*** | ***Fidelity (quality)***  ***↓***  ***Sustainability (evolution over time)*** |
| Inner setting (E) | *Organisational incentives and rewards (awards contribute to the prestige of the program)* | *Increase demand (patients act to demonstrate support/need for exercise) + Involve executive boards (feed intervention results to executive)* | *Advocacy (optimism belief in positive outcome)* | *Penetration (sub-system)* |
| Inner setting (B) | *Relative priority (competing demands for providers and organisation)* | *Remind clinicians (multiple methods - (stickers on hard-copy files, presentations, referral forms/evidence summaries at point of referral, ‘footers’ of letters)*  *Develop and distribute educational materials (manuals, how to refer)* | *Remembering and decision-making prompt at the time of decision* | *Penetration (service level)* |
| **Inner setting (E)** | **Networks and communications (personal relationships and trust create efficiencies in operations)** | **Promote network weaving (create opportunities for staff to mix across professional boundaries)** | **Social interactions and professional identity** | **Acceptability (provider)**  **↓**  **Penetration (service level)** |
| **Inner setting (E)** | ***Access to knowledge and information (one-stop-shop in survivorship)*** | ***Create new clinical teams (multi-disciplinary team)*** | ***Simplify decision-making*** | ***Penetration (service level)*** |
| **Inner setting (B)** | ***Available resources (lack of time and funding)*** | ***Access new funding (grants, fundraising, philanthropic donations)*** | ***Conditions for building material resources to develop organisational capability*** | ***Sustainability (program components)*** |
| Inner setting (B) | *Leadership engagement (leaders have low awareness of the service)* | *Champion (works across and up to executive advocating to assure resourcing for exercise)* | *Persuade to impact intention to act* | *Penetration (sub-system)*  *↓*  *Sustainability* |
| **Intervention (E)** | **Adaptability (adapt the program to suit patient needs and referrals adapted to suit provider demands)** | **Develop and implement tools for quality monitoring (exercise-specific tools to track health outcomes and compliance)**  **Promote adaptability (offer different exercise and referral processes)** | **Skill acquisition to support mastery of skills** | **Acceptability (consumer/provider)**  **↓**  **Sustainability (health outcomes)** |
| Intervention (B) | Cost (no secure funding for the program) | Involve patients’ consumers and family members (fundraising efforts) → Access new funding (funding comes from multiple sources) | Increase material resources | Sustainability (program components) |
| Outer setting (E) | Cosmopolitan (a well-connected organisation that builds capacity) | Develop resource sharing agreement (relationship with local exercise services)  Develop academic partnership (formal relationship with a university to *test and trial* program expansions | Develop material resources to build capacity | Fidelity  ↓  Sustainability (program components and evolution over time) |
| **Outer setting (E)** | **External policies and incentives (leverage COSA position statement and exercise embedded in organisation reporting required by the State)** | **Change record system (exercise added to the online electronic scheduler) → Involve executive boards (feed intervention results to executive)**  **Use mass media (emails blasts sharing COSA statements)** | **Align exercise with established goals and organisational performance targets**  **Influence intention to act** | **Penetration (sub-system)**  **↓**  **Sustainability (program component)**  **Acceptability** |
| **Outer setting (E)** | **Patient need and resourcing (patient satisfaction/needs are central to how the EBI is developed/delivered)** | **Obtain and use patient, consumer and family feedback (patient-reported outcomes (including satisfaction) collected)** | **Action planning (ongoing improvement activity)** | **Sustainability (evolution over time)** |
| **Process (E)** | ***Engaging (Champion (persistent, influential across levels of the organisation and builds patient activation))*** | ***Champion (works across and up to executive advocating to assure resourcing for exercise)***  ***Increase demand (patients act to demonstrate support/need for exercise)*** | ***Persuades to change intention to act***  ***Build optimism and belief/confidence in positive outcomes*** | ***Acceptability*** |
| **Process (E)** | **Reflecting and evaluating (review data to change practice)** | **Develop and organise a quality monitoring system (tracking system for who is using the service) → Purposely re-examine implementation (program times set for a limited duration)** | **Action planning** | **Fidelity (dose/amount)**  **↓**  **Sustainability (evolution over time)** |

| **Case Study B** | | | | |
| --- | --- | --- | --- | --- |
| **CFIR construct** | **Summary of construct extracted from framework matrix** | **Implementation strategy (ERIC)** | **Mechanism** | **Implementation outcomes** |
| Individual (B) | Individual identification with the organisation (different commitment to the organisation impacts site success) | Develop educational materials (script for co-ordinator and structured framework) | Procedural/task consistency | Fidelity (quality) |
| **Individual** | **Stage of change (program underpinned by behaviour change theory) (E)**  **(Healthcare providers act to refer, although this is not universal) (B)** | **Prepare patients to be active participants (patients supported to lead on their care)**  **Facilitate relay of clinical data to providers (regular updates provided to referral source)** | **Perceived behavioural control**  **Reinforce expected (positive) outcomes** | **Acceptability (patient)**  **↓**  **Fidelity (dose/amount)**  **Penetration (service level)** |
| **Individual (E)** | **Self-efficacy (program builds confidence in patients to exercise)**  **Healthcare staff have the confidence to discuss exercise and know where to refer** | **Intervene with patients and consumers to enhance uptake and adherence (multiple adherence strategies applied)**  **Conduct education meetings (presentations about exercise and the service); Conduct ongoing training (staff undergo exercise and cancer training course)** | **Ease and mastery of tasks**  **Knowledge (procedural)** | **Acceptability (patient)**  **↓**  **Fidelity (dose/amount)**  **Acceptability (Provider)** |
| **Inner setting (E)** | ***Learning climate (staff are supported with ongoing learning and service improvement)*** | ***Provide local technical assistance (weekly meeting to workshop implementation issues)*** | ***Action planning*** | ***Fidelity (quality)***  ***↓***  ***Sustainability (evolution over time)*** |
| **Inner setting** | **Networks and communications (hierarchy makes communication challenging (B). However, relationships between staff are strong and supportive (E))** | **Promote network weaving (provide social opportunities so staff build relationships)** | **Social interactions and professional identity** | **Acceptability (provider)** |
| **Inner setting (E)** | ***Access to knowledge and information (easy to access information because the service is established as a one-stop shop)*** | ***Use mass media (website)*** | ***Simplify decision-making*** | ***Penetration (service level)*** |
| **Inner setting (B)** | ***Available resources (lack of funding, but lean service model operates)*** | ***Access new funding (fundraising, donations)***  ***Create resource sharing agreement (special fitness centre rates and pro-bono expertise)*** | ***Builds material resources to develop organisational capabilities*** | ***Sustainability (program components)*** |
| Inner setting (E) | *Leadership engagement (leadership are highly engaged to ensure the program is a success)* | *Build a coalition (diverse partners are brought on board and sell a consistent message)* | *Optimism and group identity* | *Sustainability (program components and evolution over time)* |
| Inner setting (E) | Structural characteristics (site selection was purposeful to ensure optimisation of service) | Change service sites (hub and spoke model) | Decrease environmental barriers | Acceptability |
| **Intervention (E)** | **Adaptability (program has the flexibility to offer different exercise opportunities and referral pathways)** | **Develop and implement tools for quality monitoring (initial assessment forms, exercise sheets)**  **Promote adaptability (exercise tailored to individual needs; multiple referral pathways)** | **Skill acquisition to support mastery of skills** | **Acceptability (consumer/provider)**  **↓**  **Sustainability (health outcomes)** |
| Intervention (E) | Complexity (centralised administration/process hub) | Centralised technical assistance (centralised IT system established) | Streamline work tasks | Penetration (sub-system) |
| Intervention (B) | Cost (funding to keep costs low) | Start a dissemination organisation (a non-for-profit established) | Increase material resourcing | Sustainability (program components, evolution over time) |
| Intervention (E) | Design quality and packaging (a professional, consistent image that lends to credibility) | Use mass media (image built across different mediums) | Program identity | Acceptability |
| **Outer setting (E)** | **External policy and incentives (exercise and cancer is a policy priority of the State and a dedicated funding stream established by the State)** | **Develop academic partnership (program commenced through research trial and continues to support research)**  **Change record-keeping system (purpose-built IT system captures data for reporting) → Involve executive boards (relay outcomes to policy-makers)** | **Conditions to build capacity**  **Align exercise with established goals/performance targets** | **Fidelity (dose/amount and quality)**  **Penetration (sub-system)**  **↓**  **Sustainability (program components)** |
| **Outer setting (E)** | **Patient need and resourcing (advisory groups established that include consumers to direct patient-centred care)** | **Use advisory boards and working groups (consumers’ experiences embedded in governance structures)**  **Obtain and use patient, consumer and family feedback (patient-reported outcomes collected before clinic and used to inform service)** | **Action planning to optimise service continually** | **Sustainability (evolution over time)** |
| Process (E) | Engaging (oncology team *give permission* and can persuade people to exercise) | Identify and prepare champion (influential *expert* able to transcend levels) | Persuade to impact intention to act | Acceptability |
| ***Process (E)*** | ***Champion – (influential and respected as an expert in the field of exercise oncology)*** | ***Increase demand (patient activation)*** | ***Build optimism and belief/confidence in positive outcomes*** | ***Acceptability*** |
| Process (E) | Planning (12 months of planning before launch) | Assess for readiness and identify barriers and facilitators (planning, engaging stakeholder) | Action planning | Acceptability  ↓  Penetration (sub-system) |
| **Process (E)** | **Reflecting and evaluating (review clinical data collected through centralised IT system)** | **Develop and organise a quality monitoring system (consistent data collection and input into the IT system)** | **Action planning** | **Fidelity**  **↓**  **Sustainability (evolution over time)** |

| **Case Site C** | | | | |
| --- | --- | --- | --- | --- |
| **CFIR construct** | **Summary of construct extracted from framework matrix** | **Implementation strategy (ERIC)** | **Mechanism** | **Implementation outcomes** |
| **Individual (B)** | **Individual stage of change (for some providers discussing exercise and referral is part of their routine practice, however, they make decisions about *timing* (see *relative priority*)** | **Facilitate relay of clinical data (providers have access to patient progress through exercise)** | **Reinforces expected (positive) outcomes** | **Penetration (service level)** |
| **Individual (E)** | **Self-efficacy (some staff are confident to have conversations, whilst others do not view it as part of their role)** | **Provide ongoing training (education and training course that build from short courses through to graduate certificates)**  **Conduct education meetings (multi-disciplinary team meetings)** | **Knowledge** | **Acceptability (provider)** |
| Inner setting | Compatibility (E) (Selected parts of the organisation have fit exercise within the workflow (i.e., ‘opt-out’ referral system, linked to fitness for surgery)  (B) Where fit has not been achieved, exercise is incompatible with organisation prioritises (acute care). | Tailor strategies (ongoing change to referral processes to ensure the best fit) | Action planning | Penetration (service level) |
| ***Inner setting (E)*** | ***Learning climate – (staff undertake ongoing learning, including annual quality improvement program)*** | ***Provide clinical supervision (mentoring relationship)***  ***Nb: the exercise services’ current quality improvement program was to audit and provide feedback on triage processes (see structural characteristics)*** | ***Action planning*** | ***Fidelity (quality)***  ***↓***  ***Sustainability (evolution over time)*** |
| *Inner setting (B)* | *Relative priority (staff are busy and so make decisions about what is the most critical issue to address at the time. Exercise is not a priority for the organisation, which operates a traditional bio-medical model* | *Prepare patients and consumers to be active participants (empower patients to shift clinician behaviour)*  *Obtain and use patient, consumer and family feedback (collect feedback on response to exercise program)* | *Increase self-confidence and perceived behavioural control* | *Acceptability (patient)*  *↓*  *Penetration (service level)* |
| *Inner setting (B)* | *Tension for change (perverse incentives inhibit service growth)* | *Revise professional role (ring-fence human resources when opportunities present to grow service)* | *Build material resources* | *Sustainability (program components)* |
| ***Inner setting (E)*** | ***Access to knowledge and information (exercise contained within a program (design and packaging) makes it simpler to access information)*** | ***Create new clinical teams (exercise offered within a multi-disciplinary program)*** | ***Simplify decision making*** | ***Penetration (service level)*** |
| **Inner setting (E)** | **Networks and communication (personal relationships enhance referral to exercise)** | **Promote network weaving (build relationships between workforce to then dovetailing appointments)** | **Social interactions and professional identity** | **Acceptability (provider)**  **↓**  **Penetration (service level)** |
| ***Inner setting (B)*** | ***Available resources (differing resourcing levels impacts agile service delivery)*** | ***Develop resource-sharing agreement (formal and informal agreements in place to increase access to exercise)***  ***Access new funding (private donations, philanthropic and other sources)*** | ***Develop material resources to build the capacity of the program to meet the demand*** | ***Sustainability (program components)*** |
| Inner setting (B) | Structural characteristics (exercise in physiotherapy department)  (Insufficient space and equipment in gym and poor location) | Audit and feedback (audit triage process to improve appropriate referral of services)  Change service sites (move to a bigger site) | Reinforces desired behaviour  Environmental interactions | Penetration (sub-system)  Acceptability |
| **Intervention (E)** | **Adaptability (program adapted to suit patient preferences and referral sources)** | **Develop and implement tools for quality monitoring (templates to guide assessments and delivery)**  **Promote adaptability (different exercise EBIs to accommodate patient preference and multiple referral options for the workforce)** | **Skill acquisition to support mastery of skills** | **Acceptability (consumer/provider)**  **↓**  **Sustainability (health outcomes*)*** |
| Intervention (E) | Complexity (simplify referral processes) | Change record system (introduction of electronic medical records) | Ease, suitability and convenience (for providers) | Penetration (service system) |
| Intervention (E) | Evidence strength and quality (the accumulating evidence-based builds support for exercise) | Develop academic partnerships (run pragmatic research trials within routine care and work with the academic sector to develop education opportunities) | Skill acquisition | Fidelity (quality of care) |
| Intervention (E) | Relative advantage (exercise is beneficial for side-effects of cancer treatment, and it is the most effective mechanism to get people fit for surgery) | Develop educational materials (resources include exercise indications, exercise for fatigue and referral trial)  NB – unclear where this information is disseminated | Establishes positive beliefs about anticipated outcomes | Acceptability (provider)  ↓  Penetration (service level) |
| **Outer setting (E)** | **External policy and incentives – (COSA position statement used to legitimise exercise)**  **(Exercise EBI aligned to State cancer plan and organisational reporting required by the State)** | **Inform local opinion leaders (a respected expert who can transcend organisational hierarchy)**  ***Change record system (capture data)***  ***↓***  **Involve executive boards (bi-annual reporting)** | **Influence/shift intention to act**  **Align exercise EBI with established goals/targets** | **Acceptability**  **Penetration (sub-system)**  **↓**  **Sustainability (program components)** |
| **Outer setting (E)** | **Patient need and resourcing (advisory groups operate to drive consumer-directed care and satisfaction with exercise EBI is enhanced by tailoring to patient needs)** | **Obtain and use patient, consumer and family feedback**  **Use advisory boards and workgroups (formal groups that have a seat in strategic decision-making)** | **Action planning and engaging in ongoing improvement cycles** | **Sustainability (evolution over time)** |
| **Process** | ***Champion* *(lack of exercise champion is viewed as a barrier to 1:1 program (B). However, in other areas (youth), a champion has supported successful implementation (E)*** | ***Increase demand (use clinical data and advisory groups to demonstrate demand for service)*** | ***Persuade and build optimism*** | ***Acceptability*** |
| **Process (E)** | **Reflecting and evaluating (use data to inform resourcing needs)** | **Implement a quality monitoring system (system to capture activity, outcome and referrals)**  **NB: the outcomes of this work are documented in a record system and fed through executive boards)** | **Action planning** | **Fidelity (dose and amount)**  **↓**  **Sustainability (evolution over time)** |
| CFIR = Consolidated Framework for Implementation Research, COSA = Clinical Oncology Society Australia, EBI = Evidence-based intervention, ERIC = Expert Recommendations for Implementing Change, IT = Information Technology, **Bold** = common pathways across sites, *Italics* = Determinant is a sibling construct of another determinant in the CFIR | | | | |
